# Distinct Features of Autoimmune Gastritis in Patients with Open-Type Chronic Gastritis in Japan

**DOI:** 10.3390/biomedicines8100419

**Published:** 2020-10-14

**Authors:** Mayo Tsuboi, Ryota Niikura, Yoku Hayakawa, Yoshihiro Hirata, Tetsuo Ushiku, Kazuhiko Koike

**Affiliations:** 1 Department of Gastroenterology, Graduate School of Medicine, The University of Tokyo, Tokyo 113-8655, Japan; tsuboim-int@h.u-tokyo.ac.jp (M.T.); yhayakawa-tky@umin.ac.jp (Y.H.); kkoike-tky@umin.ac.jp (K.K.); 2 Division of Advanced Genome Medicine, The Institute of Medical Science, The University of Tokyo, Tokyo 108-863, Japan; hiratay-int@h.u-tokyo.ac.jp; 3 Department of Pathology, Graduate School of Medicine, The University of Tokyo, Tokyo 113-8655, Japan; ushikut-tky@umin.ac.jp

**Keywords:** gastritis, atrophy, microbiota, helicobacter infections, autoimmune diseases

## Abstract

In Asia, the incidences of *Helicobacter pylori* infection and gastric cancer are high, but their association with autoimmune gastritis (AIG) is unclear. This was a retrospective cohort study of patients endoscopically diagnosed with chronic gastritis between 2005 and 2017. AIG was diagnosed according to anti-parietal cell antibody positivity. Laboratory, histological findings, and gastric cancer incidence were compared between AIG and non-AIG patients. The AIG group had more females and a higher rate of thyroid disease. Serum levels of gastrin were significantly higher in AIG patients (mean 1412 and 353 pg/mL, *p* < 0.001). The endoscopic findings included a significantly higher percentage of corpus-dominant atrophy in AIG (31.67%) than in non-AIG (7.04%) patients (*p* < 0.001). Clusters of ECL cells were observed in 28% of AIG patients and 7% of non-AIG patients (*p* = 0.032). The cumulative incidence of gastric cancer at 5 and 10 years was 0% and 0.03% in the AIG group and 0.03% and 0.05% in the non-AIG group, and no significant difference in gastric cancer incidence was observed. Despite significant differences in gastrin levels between AIG and non-AIG patients, there was no evidence of an impact of AIG on the incidence of gastric cancer.

## 1. Introduction

Chronic gastritis is a common disease in Asian countries due to the high prevalence of *Helicobacter pylori (H*. *pylori)* infection. By contrast, in Western countries, the rate of *H*. *pylori* infection is much lower, and autoimmune gastritis (AIG) is the more common subtype of chronic gastritis. AIG is caused by an autoimmune process that leads to the destruction of gastric parietal cells and chief cells in the proximal stomach by specific autoantibodies, which leads to reductions in acid and intrinsic factor secretion, resulting in impaired vitamin B12 absorption and pernicious anemia [1]. Typically, impaired acid secretion is compensated by the increased activation of gastrin secretion via a negative feedback mechanism. Both enterochromaffin-like cell (ECL) hyperplasia and neuroendocrine tumor may be associated with these series of responses.

*H*. *pylori*, particularly *CagA*-positive strains, induce chronic inflammation in the stomach, which leads to the development of atrophic gastritis and intestinal metaplasia [2,3]. Atrophy and metaplasia usually arise from the distal antrum but may expand to the proximal stomach. *H*. *pylori* infection is a well-established risk factor for gastric cancer, particularly in patients with severe gastric atrophy and intestinal metaplasia [4], including in the proximal stomach. In Japan, *H*. *pylori* infection is thought to be a major cause of chronic gastritis and thus infection status is routinely measured during clinical examinations, but the prevalence of AIG in Japan is unclear. Moreover, distinguishing AIG and *H*. *pylori*-induced gastritis based on the results of routine endoscopic and histological examinations is challenging. A recent report from Japan found that nearly 20% of patients with repeated episodes of *H*. *pylori* eradication failure have anti-parietal cell antibodies (APCA), determined by serological testing, and thus potentially AIG [5]. However, the clinical characteristics and natural history of AIG in Japan are not fully understood.

Therefore, in this study, we examined the potential association between AIG and gastric cancer. Specifically, we compared the clinical features and determined the cumulative incidence of gastric cancer in the two groups.

## 2. Materials and Methods

### 2.1. Study Design and Settings and Patients

Patients examined from 2005 to 2017 with endoscopy were included in this retrospective, single-center, cohort study. Indications of upper gastrointestinal endoscopy were gastric cancer screening or gastroesophageal symptoms. The data were obtained from the Gastric Cancer Endoscopy Database of Tokyo University and the Institute for Adult Diseases, Asahi Life Foundation, a retrospectively recorded database of patients over 20, who have undergone upper gastrointestinal endoscopy. Details of the database have been reported previously [6].

As for exclusion criteria, patients with a history of gastric cancer diagnosis or gastrectomy within the past year were excluded. Patients were diagnosed with AIG for APCA titer of 1:10 or greater in serological testing, regardless of *H. pylori* infection status. The control group (non-AIG) consisted of patients who did not have APCA.

This study was approved by the Institutional Review Boards at the University of Tokyo (2058-2, 17 November 2017). Informed consent was obtained from all patients. The investigations were carried out following the rules of the Declaration of Helsinki of 1975, revised in 2013.

### 2.2. Clinical Characteristics

The following characteristics of the patients were evaluated using their medical charts: age, sex, *H*. *pylori* infection status at first visit, comorbidities, medication including low-dose aspirin, proton pump inhibitors (PPIs), hisamine-2 receptor antagonist, NSAIDs and steroids. Laboratory data, including hemoglobin, mean corpuscular volume, gastrin, vitamin B12, folic acid, serum levels of iron, and pepsinogen were measured on serum samples taken at the time of fasting. The first-time measured data were evaluated. The evaluated comorbidities included diabetes and autoimmune thyroiditis, which are associated with autoimmune diseases [7]. Medication was defined as at least 30 days of use. *H*. *pylori* infection status was evaluated based on the serum levels of *H*. *pylori*-IgG antibodies, a 13C-urea breath test, detection by gastric biopsy, stool antigen, or medical records.

### 2.3. Upper Gastrointestinal Endoscopy and Histological Examination

All upper gastrointestinal endoscopy examinations were performed by expert endoscopists. Endoscopic atrophy was evaluated using the Kimura-Takemoto classification [7], and the presence of corpus-dominant atrophy was also evaluated. In patients undergoing histological assessment, two tissue samples from the antrum and greater curvature of the corpus were assessed, a method used in our previous study [6,8]. Based on the results of histology including atrophy, neutrophil infiltration, and intestinal metaplasia, the patients were divided into three groups according to modified Updated Sydney System. Group A had no findings in either the antrum or the corpus, group B had findings in the antrum only, and group C had findings in the corpus, with or without findings in the antrum [6,8]. All histological results were assessed by an expert pathologist.

### 2.4. 16S rRNA Gene Sequencing and Analyses

16S rRNA gene sequencing was performed on patients with available gastric mucosal samples and consent for analysis.16S rRNA was isolated from biopsy samples of the greater curvature of the corpus at the same time as histological examination. Informed consent to analyze the gastric microbiome by 16S rRNA sequencing was obtained from all patients. DNA were extracted on the day of biopsy, or stored in Allprotect Tissue Reagent (Qiagen, Hilden, Germany) until extraction. The 16S rRNA gene was amplified using the QIAamp DNA mini kit (Qiagen) to extract DNA and following primers targeting the V3–4 hyper variable region: 5′-TCGTCGGCAGCGTCAGATGTGTATAAGAGACAGCCTACGGGNGGCWGCAG-3′ and 5′-GTCTCGTGGGCTCGGAGATGTGTATAAGAGACAGGACTACHVGGGTATCTAATCC-3′. Sequencing was conducted on an Illumina MiSeq System (San Diego, California, USA). The reads were filtered based on a quality score of 0.05 (*Q* = −10 log10 (*P*)) and a maximum number of ambiguities of two nucleotides. The filtered reads were clustered into operational taxonomic units (OTU) assuming 97% similarity, and each OTU was taxonomically assigned using CLC Genomics Workbench^®^ v7.5 (Qiagen, Hilden, Germany ), based on a minimum of 80% similarity with the sequences in a reference database (Greengenes Named Isolate database, 2013). From the filter-passed reads, 3000 high-quality reads per sample were selected for further analyses.

Diversity analyses were performed using CLC Genomics Workbench^®^ v7.5 (Qiagen) and SAS v9.4 (SAS Institute, Cary, NC, USA). Alpha-diversity was determined based on the total read numbers, number of OTUs, and the Simpson Index between AIG and non-AIG. Differences in alpha-diversity were analyzed using the Wilcoxon rank-sum test in comparisons between the groups. Beta-diversity was assessed based on the ten most relevant taxa and visualized in a principal coordinate analyses (PcoA) between AIG and non-AIG. Sample clustering in the beta-diversity analyses were tested using a general linear model between the groups.

### 2.5. Survival Analyses

The endpoint was gastric cancer incidence events. Gastric cancer was diagnosed by pathological evaluation of biopsy and/or resected specimens. The follow-up period was defined as the time between the first and last visits to the hospital or 31 December 2017.

### 2.6. Statistical Analyses

The full analysis set included AIG and non-AIG open-type chronic gastritis patients. The baseline characteristics of the groups were compared using a chi-square test or Fisher’s exact test as appropriate. Continuous data were evaluated using the Mann–Whitney U-test. For survival analyses, the cumulative incidence of gastric cancer risk was evaluated using the Kaplan-Meier method at 1–10 years. The incidence of gastric cancer was compared between the groups using the log rank test.

The sub-analysis set included both AIG and non-AIG patients who underwent gastric microbiota analysis. These patient characteristics were also compared using a chi-square test or Fisher’s exact test, or the Mann–Whitney U-test for continuous data. Furthermore, another analysis, comparison between PPI user and non-user, was also performed in sub-analysis set. Beta-diversity was assessed based on the ten most relevant taxa and visualized in a PcoA between PPI user and non-user.

A *p*-value < 0.05 was considered to indicate statistical significance. All statistical analyses were performed using JMP version 13 software and SAS version 9.4 (SAS Institute, Cary, NC, USA).

## 3. Results

### 3.1. Patients Characteristics

**Figure 1:**
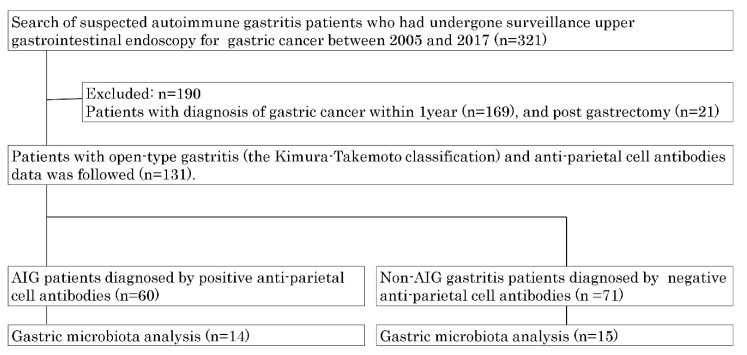
Study flow.

Of the 321 suspected AIG patients of our cohort, patients with a gastric cancer diagnosis (*n* = 169) or gastrectomy within the past year (*n* = 21) were excluded. The data of 60 AIG and 71 non-AIG open-type chronic gastritis patients were analyzed (Figure 1). The baseline characteristics of the patients are shown in Table 1. AIG patients were older (75.8 years) than non-AIG patients (73.3 years), consisted of a larger number of females, with no statistical significance. AIG patients had a statistically higher rate of thyroid diseases. Concurrent or previous *H*. *pylori* infection was confirmed in 61.66% of AIG patients and in 69.02% of non-AIG patients. An evaluation of the laboratory data showed that mean serum levels of gastrin were significantly higher in AIG patients (1412 pg/mL) than in non-AIG patients (353 pg/mL) (*p* < 0.001). Folic acid levels were also significantly higher in AIG patients. The endoscopic findings included a significantly higher percentage of corpus-dominant atrophy in AIG (31.67%) than in non-AIG (7.04%) patients (*p* < 0.001). By contrast, there were no significant differences in the histological findings of the two groups with respect to the degree of atrophy, neutrophil infiltration, and intestinal metaplasia. Clusters of ECL cells were observed in 27.59% of AIG patients and 6.67% of non-AIG patients (*p* = 0.032).

### 3.2. Gastric Microbiota

Gastric microbiota analyses were performed among 29 patients without concurrent *H. pylori* infection (14 AIG and 15 non-AIG). The baseline characteristics of the patients are shown in Table 2. The characteristics between AIG and non-AIG in patients undergoing gastric microbiota analysis did not differ compared to the full analysis group.

**Figure 2:**
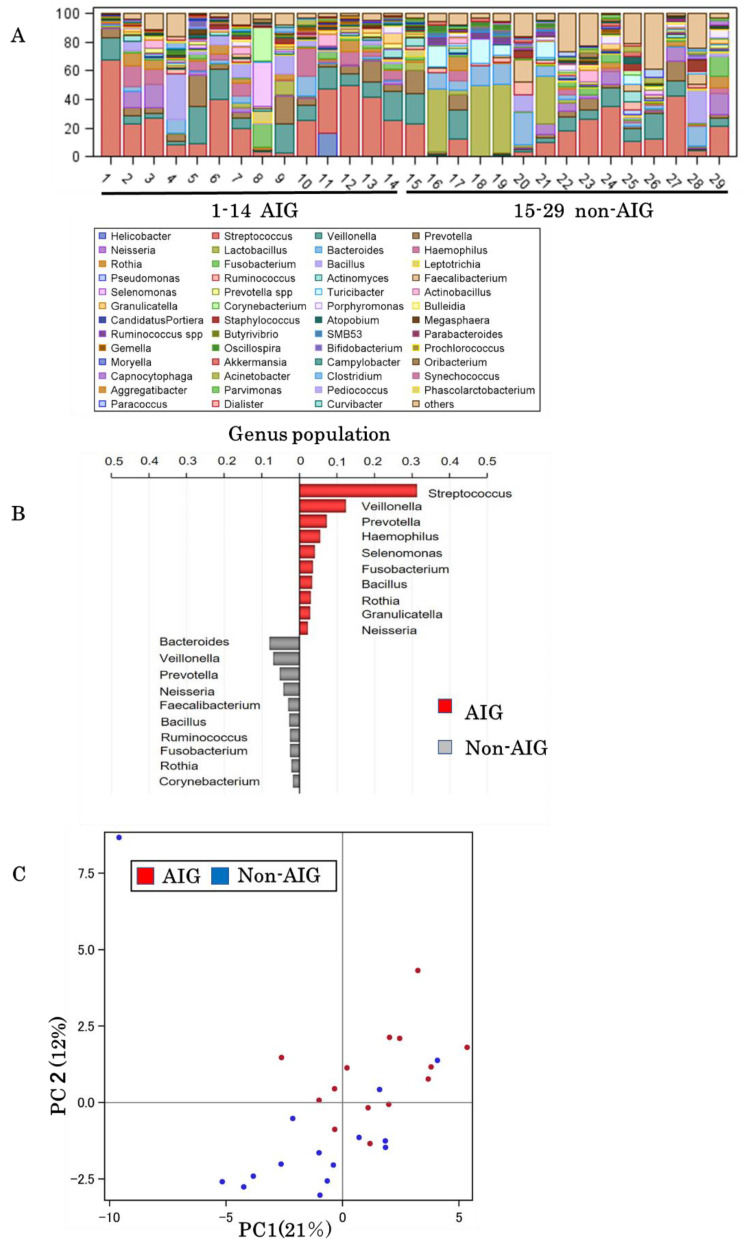
Gastric flora analysis. (**A**) Taxonomy, genus level. Comparison between AIG and non-AIG; (**B**) The ten most relevant genus taxa between AIG and non-AIG; (**C**) Principal coordinate analysis between AIG and non-AIG.

After sequencing and quality filtering, 768,168 reads were obtained (mean of 26,488 reads and 42 OTUs per sample). The AIG group had a significantly higher number of reads (mean of 32,805 vs. 20,592 reads, respectively; *p* = 0.043), non-significantly higher number of OTUs (mean of 45 vs. 39 OTUs), and non-significantly lower microbial alpha (within-sample) diversity and Simpson’s indexes. The ten most relevant microbial taxa in the biopsy samples of each group of patients were determined (Figure 2A,B). The genera Streptococcus (*p* = 0.046), Selenomonaus (*p* = 0.031), Granulicatella (*p* = 0.034), and Bacillus (*p* < 0.001) were detected in significantly higher proportions in AIG patients. Streptococcus, Haemophilus, Selenomonaus, and Granulicatella were detected only in AIG patients. The results were visualized in PCoA plots, by capturing the first two principal coordinates (PC1, 21%; PC2, 12%, Figure 2C). The microbiota composition significantly differed between the groups (R-squared = 0.858, *p* < 0.001).

**Figure 3:**
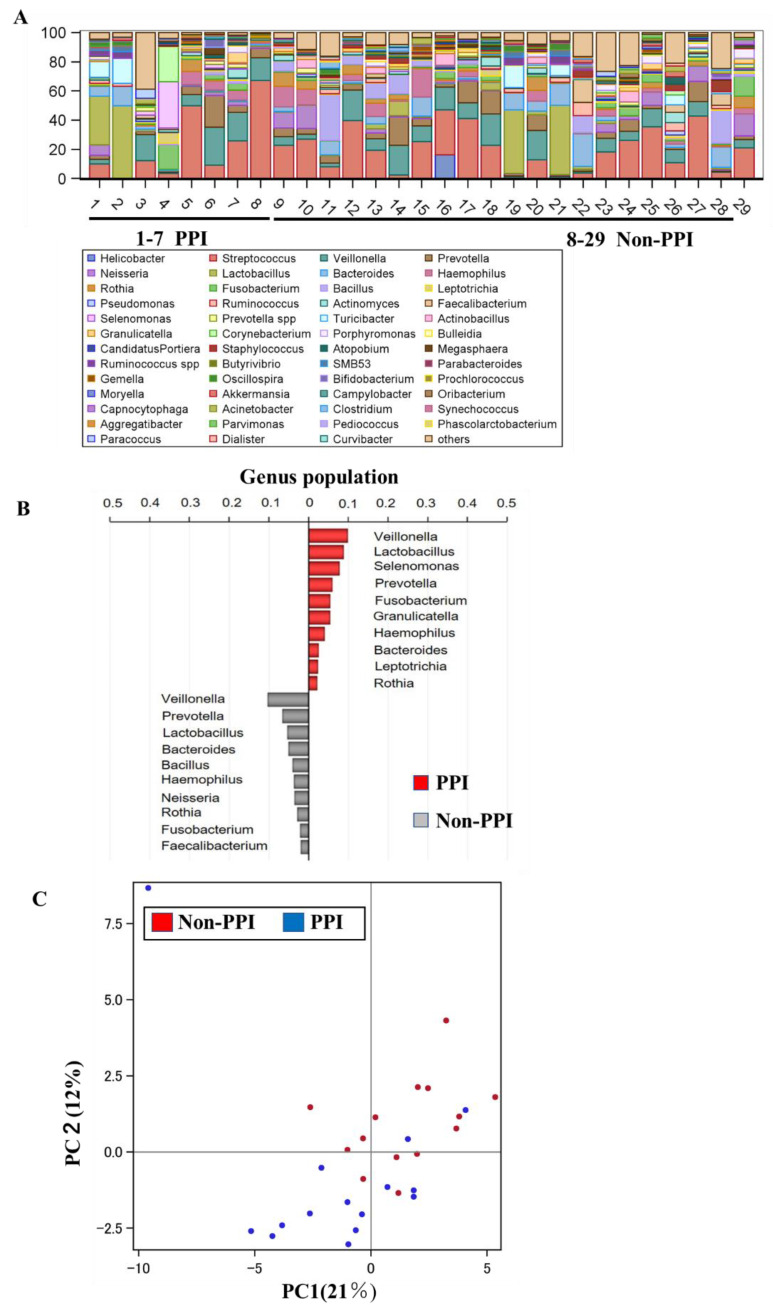
Gastric flora analysis. (**A**) Taxonomy, genus level. Comparison between proton pump inhibitor (PPI) and non-PPI; (**B**) The ten most relevant genus taxa between PPI and non-PPI; (**C**) Principal coordinate analysis between PPI and non-PPI.

Among the 29 patients (14 AIG and 15 non-AIG) for whom 16S rRNA gastric microbiota analyses were performed, there were no differences in the microbiota composition of PPI users (*n* = 7) vs. non-users (*n* = 22) (Figure 3A,C). The most frequently identified genera were Veillonella, Lactobacillus, and Selenomonas in PPI users and Veillonella, Prevotella, and Lactobacillus in non-users (Figure 3B).

### 3.3. Cumulative Gastric Cancer Incidence

**Figure 4:**
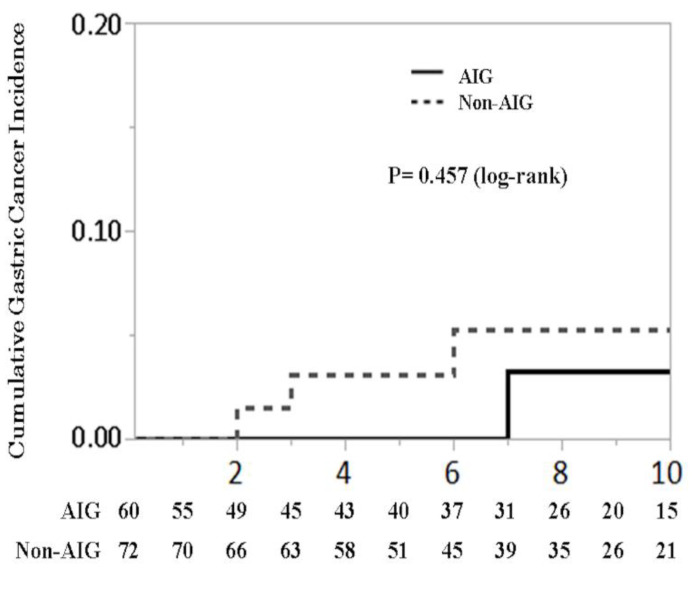
Cumulative gastric cancer incidence.

The mean follow-up period was 6.2 years (interquartile range 2.3–9.8 years) in the AIG group and 7.4 years (interquartile range 4.0–10.0 years) in the non-AIG group. During follow-up, gastric cancer was diagnosed in one patient from the AIG group and in three patients from the non-AIG group. The cumulative incidence of gastric cancer in the AIG group was 0% at 1 year, 0% at 3 years, 0% at 5 years, and 0.03% 10 years. In the non-AIG group was 0% at 1 year, 0.03% at 3 years, 0.03% at 5 years, and 0.05% at 10 years (*p* = 0.457, log-rank) (Figure 4).

## 4. Discussion

We found that AIG patients had higher serum levels of gastrin and a higher proportion of ECL cell hyperplasia than non-AIG patients. However, there were no significant differences in the rate of gastric cancer development.

Higher gastrin levels in AIG patients (than in non-AIG patients) has been reported in a previous study [5]. A loss of parietal cells, which occurs in AIG, reduces gastric acid secretion and induces an increase in serum levels of gastrin via a negative feedback mechanism [7]. Prolonged gastric acid suppression by PPI has potential to limit the recovery of gastric atrophy [9]. Hypergastrinemia, in turn, induces an increase in the number of gastric ECL cells, which express gastrin receptors. A previous study in animal models have reported that hypergastrinemia may increase the risk for neuroendocrine tumors (NETs) [10]. However, in our study, while ECL cell hyperplasia occurred more frequently in AIG patients, neither group developed NETs during the observation period.

The genera *Streptococcus* and *Haemophilus* were detected significantly more often in AIG patients than in non-AIG patients, in agreement with a previous result obtained using culture-based methods [5]. In addition, these findings were consistent with recent 16S rRNA-based study including AIG patients [11]. In contrast, the presence of *Klebsiella*, identified in one of our patients with AIG and in culture-based studies [5], was not reported in the recent 16S rRNA-based study [11]. The difference might be due to sample selection bias among these studies might have played a role, with differences in patient characteristics including pepsinogen I and II levels as well as the degrees of endoscopically determined atrophy and pathological intestinal metaplasia.

Interestingly, the gastric microbiota did not differ between PPI users and non-users in our study (Figure 3), in contrast to the findings of a previous study [12]. This discrepancy can perhaps be attributed to the small number of PPI users in our study (*n* = 7) and to differences in the dose and duration of PPI use. In addition, most of our patients had a history of H. pylori eradication, and the effects of PPIs on the gastric microbiome may be different from those in healthy individuals.

Previous studies have suggested that hypergastrinemia and an altered gastric microbiome are associated with increased risk for gastric malignant diseases. However, in our AIG patients, there was no significant association between AIG and a higher gastric cancer incidence versus non-AIG patients. This finding can be explained as follows. First, 60% of our AIG patients also had concurrent or previous *H*. *pylori* infection, which in itself is a strong risk factor for gastric cancer. Second, missing data in our study might have resulted in differences compared to other studies. However, an increased risk for ECL cell hyperplasia was determined in our AIG versus non-AIG patients (*p* = 0.028). Our data and those of previous studies suggest that gastrin differentially influences the development of gastric cancers and NETs [4], with the former likely originating from gastric stem cells and the latter from ECL cells [13], although the two cell types express the same gastrin receptor. Data on the cumulative gastric cancer risk and NETs are still limited, and further studies are needed to evaluate these associations.

Our study is the first to compare the cumulative gastric cancer incidence of AIG and non-AIG patients, including those with gastritis related to *H*. *pylori*. However, it also had several limitations. First, it was a retrospective single-center study. Second, the diagnosis of AIG was limited to endoscopically determined atrophy and serological APCA levels; the levels of anti-intrinsic factor antibody were not determined. By missing AIG patients with positive anti-intrinsic factor antibody and negative APCA levels, the incidence of AIG may have been underestimated. In addition, 61.6% of our AIG patients had concurrent or previous *H*. *pylori* infection. Although the *H*. *pylori* infection status did not differ between AIG and non-AIG patients, in the former, the number with disease not related to *H*. *pylori* was unclear. Third, our gastric microbiota analyses were limited to selected patients because consents and gastric mucosal samples could not be obtained for all patients. A larger and more comprehensive study is required to better understand the characteristics of AIG in countries with high *H*. *pylori* infection rates.

In conclusion, AIG patients had higher serum levels of gastrin and differences in their gastric microbiota compared to non-AIG patients. Our data also suggest that, in patients with high *H*. *pylori* infection rates, the presence of AIG and hypergastrinemia together with an altered gastric microbiome has a more direct association with ECL cell hyperplasia than with gastric cancer.

## Figures and Tables

**Table 1:** Characteristics between autoimmune gastritis (AIG) and non-AIG in patients.

	AIG (*n* = 60)	Non-AIG (*n* = 71)	*p* Value
Mean age	75.80 ± 9.59	73.32 ± 9.58	0.143
Sex, female (%)	30 (50.00)	26 (36.62)	0.123
*H. pylori* infectious states (%)			
Positive	17(28.33)	20 (28.17)	0.662
Eradicated	20 (33.33)	29 (40.85)	
Negative	18 (30.00)	15 (21.13)	
Unknown	5 (8.33)	7 (9.86)	
Comorbidity (%)			
Diabetes mellitus	13 (21.67)	18 (25.35)	0.621
Chronic heart disease	11 (18.33)	15 (21.13)	0.690
Ischemic heart disease	8 (13.33)	11 (15.49)	0.726
Thyroid diseases	15 (25.00)	6 (8.33)	0.009
Collagen diseases	4 (6.67)	1 (1.39)	0.106
Medication (%)			
Low dose aspirin	6 (10.00)	8 (11.27)	0.815
Proton pump inhibitors	20 (33.33)	20 (28.17)	0.523
Histamine 2 receptor antagonist	19 (31.67)	16 (22.54)	0.239
Non-steroidal anti-inflammatory drugs	12 (20.00)	12 (16.90)	0.648
Steroids	3 (5.00)	9 (12.68)	0.129
Laboratory data *			
Hemoglobin, g/dL	10.45 ± 2.67	10.16 ± 2.71	0.530
Mean corpuscular volume, fL	88.99 ± 7.26	88.16 ± 6.46	0.489
Gastrin, pg/mL	1412 ± 2023	353 ± 471	<0.001
Vitamin B12, pg/mL	273 ± 276	520 ± 473	0.074
Folic acid, ng/mL	11.33 ± 5.22	6.93 ± 2.80	0.016
Iron, μg/dL	61.98 ± 37.70	60.47 ± 39.00	0.848
Pepsinogen I, ng/mL	37.30 ± 41.25	63.82 ± 63.27	0.154
Pepsinogen II, ng/mL	17.92 ± 12.58	20.26 ± 6.21	0.695
Pepsinogen I / II, ng/mL	1.96 ± 1.99	3.36 ± 2.18	0.069
Endoscopic findings			
Atrophy, O-I/O-II/O-III (%)	14/14/32 (23.33/23.33/53.33)	23/21/27 (32.39/29.58/38.03)	0.211
Corpus dominant atrophy (%)	19 (31.67)	5 (7.04)	<0.001
Histological findings ^†,^* (%)			
Atrophy, Group A/B/C	4/4/18 (15.38/15.38/69.23)	6/3/18 (22.22/11.11/66.67)	0.769
Neutrophils infiltration, Group A/B/C	12/2/13 (44.44/7.41/48.15)	12/3/16 (38.71/9.68/51.61)	0.889
Intestinal Metaplasia, Group A/B/C	17/1/13 (54.84/3.23/41.94)	20/6/9 (57.14/17.14/25.71)	0.116
ECL (%)	8 (27.59)	2 (6.67)	0.032

Abbreviations: AIG, autoimmune gastritis; *H. pylori*, *Helicobacter pylori*; O-, Open type. ^†^ Group A had no histological findings in either the antrum or the corpus, Group B had histological findings in the antrum only, and Group C had histological findings in the corpus, with or without presence in the antrum. * missing data included ± presented standard deviation. Parentheses presented percentage.

**Table 2:** Characteristics between AIG and non-AIG in patients undergoing gastric microbiota analysis.

	AIG (*n* = 14)	Non-AIG (*n* = 15)	*p* Value
Mean age	71.1 ± 10.17	69.4 ± 9.71	0.667
Sex, female	8 (57.14)	6 (40.00)	0.356
*H. Pylori* infectious states (%)			
Positive	0 (0.00)	0 (0.00)	0.029
Eradicated	10 (71.43)	15 (100.00)	
Negative	4 (28.57)	0 (0.00)	
Unknown	0 (0.00)	0 (0.00)	
Comorbidity (%)			
Diabetes mellitus	2 (14.29)	1 (6.67)	0.498
Chronic heart disease	1 (7.14)	1 (6.67)	0.960
Ischemic heart disease	2 (14.29)	1 (6.67)	0.501
Thyroid diseases	7 (50.00)	0 (0.00)	0.002
Collagen diseases	2 (14.29)	0 (0.00)	0.129
Medication (%)			
Low dose aspirin	0 (0.00)	0 (0.00)	0
Proton pump inhibitors	5 (35.71)	4 (26.67)	0.599
Histamine 2 receptor antagonist	1 (7.14)	3 (20.00)	0.316
Non-steroidal anti-inflammatory drugs	3 (21.43)	0 (0.00)	0.002
Steroids	0 (0.00)	2 (13.33)	0.157
Laboratory data *			
Hemoglobin, g/dL	12.09 ± 1.89	12.89 ± 1.75	0.246
Mean corpuscular volume, fL	89.73 ± 7.38	90.81 ± 5.97	0.666
Gastrin, pg/mL	3507 ± 2011	381 ± 385	0.020
Vitamin B12, pg/mL	150 ± 88	846	<0.001
Folic acid, ng/mL	10.82 ± 3.84	3.7	0.103
Iron, μg/dL	74.45 ± 39.77	53.20 ± 22.59	0.289
Pepsinogen I, ng/mL	23.2 ± 35.47	100.8 ± 79.27	0.015
Pepsinogen II, ng/mL	13.25 ± 6.93	32.86 ± 28.76	0.044
Pepsinogen I/ II, ng/mL	1.64 ± 2.50	3.12 ± 1.73	0.255
Endoscopic findings			
Atrophy, O-I/O-II/O-III (%)	1/0/13 (7.14/0/92.86)	5/4/6 (33.33/26.67/40.00)	0.01
Corpus dominant atrophy (%)	10 (71.43)	1 (6.67)	<0.001
Histological findings ^†,^* (%)			
Atrophy Group A/B/C	1/0/12 (7.69/0.00/92.31)	3/2/10 (20.00/13.33/66.67)	0.217
Neutrophils infiltration Group A/B/C	4/2/7 (30.77/15.38/53.85)	6/2/7 (40.00/13.33/46.67)	0.879
Intestinal Metaplasia Group A/B/C	6/0/8 (42.86/0.00/57.14)	7/5/3 (46.67/33.33/20.00)	0.026
ECL (%)	7 (58.85)	0 (0.00)	0.001

Abbreviations: AIG, autoimmune gastritis; *H. pylori*, *Helicobacter pylori*; O-, Open type. ^†^ Group A had no histological findings in either the antrum or the corpus, Group B had histological findings in the antrum only, and Group C had histological findings in the corpus, with or without presence in the antrum. * missing data included. ± presented standard deviation. Parentheses presented percentage.

## References

[1] Neumann W.L., Coss E., Rugge M., Genta R.M. (2013). Autoimmune atrophic gastritis--pathogenesis, pathology and management. Nat. Rev. Gastroenterol. Hepatol..

[2] Hayakawa Y., Sethi N., Sepulveda A.R., Bass A.J., Wang T.C. (2016). Oesophageal adenocarcinoma and gastric cancer: Should we mind the gap?. Nat. Rev. Cancer.

[3] Shichijo S., Hirata Y., Niikura R., Hayakawa Y., Yamada A., Ushiku T., Fukayama M., Koike K. (2016). Histologic intestinal metaplasia and endoscopic atrophy are predictors of gastric cancer development after Helicobacter pylori eradication. Gastrointest. Endosc..

[4] Hayakawa Y., Chang W., Jin G., Wang T.C. (2016). Gastrin and upper GI cancers. Curr. Opin. Pharmacol..

[5] Furuta T., Baba S., Yamade M., Uotani T., Kagami T., Suzuki T., Tani S., Hamaya Y., Iwaizumi M., Osawa S. (2018). High incidence of autoimmune gastritis in patients misdiagnosed with two or more failures of H. pylori eradication. Aliment. Pharmacol. Ther..

[6] Niikura R., Hayakawa Y., Hirata Y., Konishi M., Suzuki N., Ihara S., Yamada A., Ushiku T., Fujishiro M., Fukayama M. (2018). Distinct Chemopreventive Effects of Aspirin in Diffuse and Intestinal-Type Gastric Cancer. Cancer Prev. Res..

[7] Kulnigg-Dabsch S. (2016). Autoimmune gastritis. Wien. Med. Wochenschr..

[8] Shichijo S., Hirata Y., Sakitani K., Yamamoto S., Serizawa T., Niikura R., Watabe H., Yoshida S., Yamada A., Yamaji Y. (2015). Distribution of intestinal metaplasia as a predictor of gastric cancer development. J. Gastroenterol. Hepatol..

[9] Niikura R., Hayakawa Y., Hirata Y., Ogura K., Fujishiro M., Yamada A., Ushiku T., Konishi M., Fukayama M., Koike K. (2019). The Reduction in Gastric Atrophy after Helicobacter pylori Eradication Is Reduced by Treatment with Inhibitors of Gastric Acid Secretion. Int. J. Mol. Sci..

[10] Eissele R., Patberg H., Koop H., Krack W., Lorenz W., McKnight A.T., Arnold R. (1992). Effect of gastrin receptor blockade on endocrine cells in rats during achlorhydria. Gastroenterology.

[11] Parsons B.N., Ijaz U.Z., D’Amore R., Burkitt M.D., Eccles R., Lenzi L., Duckworth C.A., Moore A.R., Tiszlavicz L., Varro A. (2017). Comparison of the human gastric microbiota in hypochlorhydric states arising as a result of Helicobacter pylori-induced atrophic gastritis, autoimmune atrophic gastritis and proton pump inhibitor use. PLoS Pathog..

[12] Jackson M.A., Goodrich J.K., Maxan M.E., Freedberg D.E., Abrams J.A., Poole A.C., Sutter J.L., Welter D., Ley R.E., Bell J.T. (2016). Proton pump inhibitors alter the composition of the gut microbiota. Gut.

[13] Waldum H.L., Fossmark R. (2019). Role of Autoimmune Gastritis in Gastric Cancer. Clin. Transl. Gastroenterol..

